# Antimicrobial Peptides from Photosynthetic Marine Organisms with Potential Application in Aquaculture

**DOI:** 10.3390/md21050290

**Published:** 2023-05-08

**Authors:** José María García-Beltrán, Marta Arizcun, Elena Chaves-Pozo

**Affiliations:** 1 Immunobiology for Aquaculture Group, Department of Cell Biology and Histology, Faculty of Biology, Campus Regional de Excelencia Internacional “Campus Mare Nostrum”, University of Murcia, 30100 Murcia, Spain; josemaria.garcia4@um.es; 2 Physiology and Welfare of Marine Species Group (PHYSIS), Centro Oceanográfico de Murcia, Instituto Español de Oceanografía (COMU-IEO), CSIC, Puerto de Mazarrón, 30860 Murcia, Spain; marta.arizcun@ieo.csic.es

**Keywords:** antimicrobial peptides, algal peptides, aquaculture, antimicrobial, antioxidant, immunoregulatory

## Abstract

Aquaculture production is at a record level and is estimated to increase in the coming years. However, this production can be negatively affected by infectious diseases produced by viruses, bacteria, and parasites, causing fish mortality and economic losses. Antimicrobial peptides (AMPs) are small peptides that may be promising candidates to replace antibiotics because they are the first line of defense in animals against a wide variety of pathogens and have no negative effects; they also show additional activities such as antioxidant or immunoregulatory functions, which makes them powerful alternatives for use in aquaculture. Moreover, AMPs are highly available in natural sources and have already been used in the livestock farming and food industries. Photosynthetic marine organisms can survive under all kinds of environmental conditions and under extremely competitive environments thanks to their flexible metabolism. For this reason, these organisms represent a powerful source of bioactive molecules as nutraceuticals and pharmaceuticals, including AMPs. Therefore, in this study we reviewed the present knowledge about AMPs from photosynthetic marine organism sources and analyzed whether they could be suitable for use in aquaculture.

## 1. Introduction

Aquaculture is one of the most economically important food sectors worldwide. Its production is at a record level, and the sector will play a crucial role in supplying food in the coming years, estimated to increase by another 14% by 2030 [1]. However, aquaculture is affected by infectious diseases produced by viruses, bacteria, protists, and metazoans, which are common in the ocean but seem more problematic at fish farms, where the high stocking density and stressful conditions are perfect for opportunistic infections. Therefore, marine diseases represent a major cost for aquaculture, causing losses of billions of dollars each year [2]. They can affect the economic value of cultivated species by causing chronic stress, decreasing growth, reducing immunity, and increasing the mortality of fish [2,3,4]. In order to avoid or control disease outbreaks, fish farms must invest in methods to prevent and treat infections, which often involve a high cost. Immunostimulants and vaccines against viruses and bacteria are the only allowable options due to the negative effects of antibiotics on fish and the environment [2]. In addition, adverse drug reactions can occur in humans after consuming aquatic products with antibiotic residues [5,6,7]. Within this framework, antimicrobial peptides (AMPs) have attracted attention as promising candidates to replace antibiotics in order to combat the increasing number of multidrug-resistant pathogenic bacteria while avoiding the negative effects on aquaculture [8].

AMPs are small peptides that are generally formed by fewer than 50 amino acids [9,10], weigh less than 10 kDa [11,12], and have a wide range of antimicrobial activities [10,13]. AMPs are phylogenetically ancient and highly conserved molecules that have a crucial role in the immune system of living organisms as the first line of defense against a broad variety of pathogens such as Gram− and Gram+ bacteria, viruses, fungi, and parasites [7,9,10,11,12,13,14]. Their characteristics and structure allow them to have a wide variety of mechanisms of action to prevent the development of antimicrobial resistance [7,9,10,12,15]. In contrast to antibiotics, AMPs can target the entire surface of cell membranes [12], are less likely to be targeted by proteases of microorganisms as they have unique protease-binding epitopes, and are active at quite low minimum inhibitory concentrations (MICs) [10]. That is why AMPs have already been used in the livestock farming and food industries, as they can improve animal immunity and increase production performance, and they can also be used as food preservatives in the production and storage of dairy products, meat products, and canned food as they inhibit the growth of many microorganisms [7]. In fact, as food preservatives, AMPs are well digested and hydrolyzed, have no toxic side effects, have good solubility, are active under acidic conditions, and are safer than most preservatives [7]. So, although certain aspects have to be studied before AMPs can be used in aquaculture, they are a promising option to achieve the Blue Transformation initiative proposed by the Food and Agriculture Organization (FAO) that aims for economically and environmentally sustainable aquaculture and better food security [1].

Regarding the literature, the effects of algae as additives in fish diets [16,17] and the biological activity of algal AMPs [15,18] have been reviewed. In this study, we carried out a combined analysis of the biological activities of different AMPs from photosynthetic marine organisms with the potential for use in aquaculture. A selection of approximately 200 articles published between 1996 and 2022 retrieved through PubMed^®^ searches, including the terms “algae” or “antimicrobial peptides” or “biological activities” or “aquaculture”, were examined for the purpose of this review.

## 2. Classification of Antimicrobial Peptides

Generally, AMPs are small, positively charged, amphipathic molecules that have both hydrophilic and hydrophobic surfaces [11]. AMPs are classified based on multiple features due to their huge diversity (for review, see [19] and the Antimicrobial Peptide Database (APD), https://aps.unmc.edu/ accessed on 17 March 2023). In general, the main classifications are based on source, activity, or structural characteristics [19]. AMPs are produced by bacteria, fungi, plants, and animals, including invertebrates, amphibians, fish, birds, and mammals [7,19]. The APD uses 25 biological activities to classify around 3569 AMPs from all life kingdoms [20]. Zhang et al. [7] classified them according to their structural features, based on length, hydrophobic amino acid content, charge, and secondary structure. In terms of length, approximately 90% of AMPs present fewer than 50 amino acids, and most functional peptides have fewer than 30 amino acids. Regarding hydrophobicity, most AMPs have a hydrophobic amino acid content of 50%. Hydrophobicity is crucial in their antimicrobial activity, as it allows them to interact with the cell membranes of pathogens. As to their charge, almost 90% of AMPs are cationic, with a net positive charge of +1 to +13 due to arginine and lysine residues, enabling them to bind to negatively charged bacterial cell membranes [7]. Finally, according to their secondary structure, AMPs are classified as linear or cyclic, containing α-helical, hairpin-like β-sheet, or mixed α-helical/β-sheet structures stabilized by two, three, or four disulfide bonds between cysteine residues. Moreover, AMPs present an abundance of amino acids such as proline, tryptophan, histidine, and glycine [7,9,11,21].

## 3. Mode of Action of Antimicrobial Peptides

Understanding the main differences between prokaryotic and eukaryotic cell membrane structures, including the absence of cholesterol and a greater presence of anionic lipids in prokaryotic membranes [9], is key to understanding the preferential activity of AMPs against bacteria and their low toxicity against eukaryotic cells [9,11]. Regarding the mode of action, great diversity and specificity among particular AMP–bacteria couplings have been described [9]. First of all, there is electrostatic binding between the positively charged AMPs and the negatively charged lipids present in the membranes of microorganisms. Secondly, the secondary and amphiphilic structure of AMPs helps them to insert into or cross through the cell membrane. Thus, the α-helix domains of AMPs are related to their linkage with the phospholipid bilayer, while the β-folded domains are important for stability and crossing the cell membrane [7]. Afterwards, the antimicrobial activity of AMPs may or may not involve membrane damage [7,9,11,12]. Regarding their activity in membrane damage, different models of action have been postulated [7,9,11,12]. In the aggregation model, AMPs create irreversible membrane pores or ion channels, but in the carpet and agglutination models they can also induce membrane disruption and cytoplasm efflux, which in turn results in osmotic lysis of the cell [7,9,11,12]. On the other hand, AMPs that are able to cross the bacterial phospholipid bilayer subsequently inhibit bacterial growth and metabolism by disrupting cellular processes when binding intracellular components [7,9,10,11,12] or block the energy production and transfer pathway, triggering cell death [12].

In contrast to the bactericidal activity of AMPs, little knowledge is available about the molecular mechanism of their antiviral activity, and much less is available about their parasitic activity [22]. AMPs may have stage-specific action on different viruses at extracellular or intracellular stages of the viral cycle [23]. Regarding their action at extracellular stages, some AMPs can block or reduce the infectivity of viruses [24] directly, by disrupting the viral envelope [25,26,27,28], causing disintegration of the viral capsid or agglutination of viral particles [25,26,27,28,29], or indirectly, by competing for protein link sites in host cell membranes [25,30]. In any case, they prevent the penetration of viruses into host cells and subsequent replication [27,28]. The AMPs that act intracellularly can also selectively inhibit viral protein synthesis or the release of viral particles by interfering with the intracellular transport of capsid proteins to the cell surface and preventing the insertion of virions into the cell membrane [31].

Regarding the antiparasitic mode of action of AMPs, it seems to be based on their capability to distinguish the lipid composition of plasma membranes [32]. The plasma membranes of lower eukaryotes (parasites), compared to vertebrates (hosts), have anionic phospholipids at their outer leaflet, as well as different levels of sterols and plasma membrane potential, which allow mechanisms of action based on disrupting membranes, as previously described for AMPs (for review, see [32]).

## 4. Antimicrobial Peptides from Marine versus Terrestrial Sources

In addition to the abiotic condition of the oceans, including pressure, temperature range, light penetration, oxygen and salt concentration, light radiation exposure, and a wide range of microbes (bacteria and viruses), marine organisms naturally produce chemically diverse bioactive compounds that are considered essential for the discovery and development of new pharmaceutical drugs [9,11,12,33,34,35,36,37]. Marine AMPs have a different structure than terrestrial AMPs because they have had to adapt to higher concentrations of free ions in the marine environment as a result of the high salt concentration [9]. Therefore, marine AMPs present various post-translational modifications, which are required for their stability and at the same time allow them to interact with target surfaces [9]. Among these post-translational modifications are bromination, chlorination, C-terminal amidation, a high content of certain amino acids (such as phenylalanine and arginine), the modification of single amino acids (such as 3-methylisoleucine), and the presence of D-amino acids [9]. Due to these modifications, marine AMPs have a different isoelectric point (pI), secondary structure, hydrophobicity, and amphipathicity than AMPs from terrestrial sources [9]. All of these features have attracted the interest of the pharmaceutical sector, and some marine peptides have already been approved by the United States Food and Drug Administration (FDA) as pharmaceuticals for human medicine [15,21]. However, the huge biodiversity in the oceans leads to the supposition that the majority of marine AMPs are still unknown.

## 5. Antimicrobial Peptides from Photosynthetic Organisms

Photosynthetic marine organisms include algae (micro- and macroalgae) and bacteria (cyanobacteria). Cyanobacteria, or blue-green algae, are a diverse group of prokaryotes that can produce huge numbers of AMPs through either ribosomal or non-ribosomal biosynthesis [15,38,39]. Non-ribosomal peptide (NRP) synthesis is carried out by NRP synthetases (NRPSs), which are organized in an array of modules constituting a separate functional entity that adds new building blocks to the polypeptide chain. In addition, each module can add modifications to the monomer [39,40,41,42]. Together with monomer modifications carried out by the additional domains from some NRPS modules, the incorporation of polyketide moieties by polyketide synthases (PKSs) increases the number of modifications, which in turn increases the variability of peptides [15,33,39,40,41,42,43].

These post-translational modifications are well documented in reviews by Rivas and Rojas [39] and Ribeiro et al. [40]. According to these reviews, cyclization between the amino and carboxylic terminals through an amide bond results in the formation of head-to-tail, chemically stable lactone, ether, thioether, and disulfide bonds, among others. This cyclization confers resistance to exopeptidase degradation, extends the half-life, and improves passive diffusion of the peptide across biological membranes. Moreover, cyclic AMPs can incorporate polyketide moieties or heterocycles. Peptides can also incorporate carbohydrates and lipids. Among the lipids, saturated and unsaturated fatty acids (lipo- and depsi-peptides) [9], cholesterol, phospholipids, and glucolipids are incorporated through an amide, thioether, or ester bond [30,40]. This lipidation contributes to the acquisition of the biologically active conformation of the peptide, increases its overall hydrophobicity, and provides the capability of anchoring to the target cell membrane and subsequent disruption of the phospholipid bilayer. The hydrophobicity of the peptides is also increased by N-methylation, which also confers rigidity to cyclic AMPs [39]. The presence on AMPs of D-amino acids instead of L-amino acids (stereoisomerism) makes peptides resistant to proteolysis and helps in the acquisition of an optimal conformation for their biological function. Finally, heterocyclation, which is the cyclation of serine, threonine, or cysteine residues, gives rise to oxazole (Ozl), methyl-oxazole (mOzl), thiazole (Tzl), methyloxazoline (mOzn), and thiazoline (Tzn) rings and provides greater rigidity to the peptide scaffold, which might be important in redox reactions [39].

Although the identification and characterization of the biological activity of discrete peptides is a quite novel field of study, a huge number of cyanobacterial AMPs with many interesting biological activities and post-translational modifications have been discovered (for review, see [38,39,40,41,42,43,44,45]). Due to the great variability of cyanobacterial AMPs, in this study we only included AMPs that display biological activities that could be important in aquaculture, such as antimicrobial, antioxidant, and immunoregulatory activities. Peptides with antitumor, anticoagulant, antihypertensive, antidiabetic, antiobesity, or antifatigue activities, among others, were not considered. Moreover, as the main aim of this review was to gain an overall view of AMPs suitable for nutraceutical and pharmaceutical use in aquaculture, and taking into account that simplicity is an important feature of high industrial production of nutraceuticals, we focused on AMPs with slight post-translational modifications, such as cyclic peptides and peptides with Tzl and Ozl groups. Table 1 summarizes the cyanobacterial peptides that are suitable for use in disease control in aquaculture, and their most relevant activities.

Contrary to cyanobacteria, which present ribosomal and non-ribosomal biosynthesis, eukaryotic algae only have ribosomal peptide (RP) biosynthesis capability. Peptides from micro- and macroalgae are encoded by genes and synthetized by the ribosomal machinery. Therefore, they present the 20 proteinogenic amino acids with few post-translational modifications [15,39,43]. As in the case of cyanobacterial AMPs, only those that have activities relevant to aquaculture were considered. Table 2 and Table 3 summarize the most interesting peptides from microalgae and macroalgae, respectively, for use in aquaculture.

## 6. The Use of Antimicrobial Peptides in Fish Aquaculture

Infectious diseases caused by viruses, bacteria, protists, and metazoans are among the main problems of modern aquaculture. The high population density and stressful conditions at fish farms are perfect for opportunistic infections [2]. Due to the negative effects of antibiotics on fish and the environment [2,5,6,7], immunostimulants are the best alternatives to these compounds [2]. In this sense, AMPs are promising candidates to replace antibiotics for combating infectious diseases and mitigating their negative effects in aquaculture [8]. AMPs play a crucial role in the immune system of living organisms as part of the first line of defense against a wide range of pathogens, including bacteria, viruses, fungi, and parasites [7,9,10,11,12,13,14]. AMPs have already been used in the animal husbandry and food industries, and it has been shown that they can enhance animal immunity and increase production performance [7]. For their part, algae play a key role as primary producers and as the basis of the marine food chain [98], and they are a valuable source of these compounds. For these reasons, AMPs from micro- and macroalgae can be valuable resources to prevent or treat infectious diseases that can be generated in fish farms without any negative effects on fish or the environment, and they can be good candidates as ingredients for functional fish diets.

Although AMPs are a promising alternative to antibiotics, there are certain aspects that have to be considered when using them as pharmaceuticals. Natural AMPs often lack efficacy compared with conventional antibiotics [9], as their activity and stability under certain conditions are sometimes suboptimal and difficult to regulate [7,9,40]. Moreover, due to their heterogeneous structure, isolating or chemically synthesizing them is difficult and expensive [9,11,40].

However, although AMPs present some disadvantages, it is possible to enhance their efficacy, activity, and stability by applying chemical modifications [9,40]. Given their amphipathic nature, modifications that allow reduced hydrophobicity while maintaining sufficient positive charge are essential to decrease interactions between AMPs and mammalian cells and increase the targeting of bacterial cells [9]. On the other hand, cyclization of linear peptides has been considered one of the most promising methods to enhance the pharmacological activity of AMPs. A cyclic structure provides a more rigid secondary structure and stability against hydrolyzation by exopeptidases in comparison to linear peptides [40]. Regarding the cost of synthesis, AMP genes can be cloned into vectors that allow efficient expression, increasing production yield and reducing costs [7]. Considering the disadvantages of using AMPs and the possible solutions, AMPs are good candidates for use in aquaculture, although further research on their synthesis and application is necessary.

Regarding the possible use of AMPs in aquaculture, it is important to note that besides their antibacterial, antifungal, antiviral, and antiparasitic activity, AMPs also present antioxidant [12] and immune modulatory activity, linking innate and adaptive immunity and triggering effective elimination of pathogens [10,11,12,13,14,33,99]. As we can see summarized in the tables, AMPs present powerful antioxidant activity through radical scavenging activity and increasing the activity of antioxidant enzymes, which is crucial for the stressful situations that can occur at fish farms. Moreover, AMPs can regulate the differentiation and activation of relevant immune cells such as macrophages, monocytes, dendritic cells, lymphocytes, cluster of differentiation (CD)4+ and CD8+ T cells and B cells, and natural killer (NK) cells [12,14] and have a role in cytokine and chemokine production and release, antigen presentation, inflammatory responses [9,10], endotoxin neutralization [11], and wound healing [11,14,99]. These molecules may also present pro-inflammatory [14,99] and anti-inflammatory [10,11,12] activity, as they can activate the inflammatory process through a wide range of molecular mechanisms, such as activation of the nuclear factor kappa B (NF-kB) and mitogen-activated protein kinase (MAPK)-dependent pathways [14], but they can also avoid an excessive inflammatory response by regulating cytokine release from macrophages and monocytes [12].

## 7. Algae as a Vehicle for Treating Fish with Antimicrobial Peptides

In general, in aquaculture, feeding strategies are considered that are the most suitable for treating aquatic animals. Apart from their nutritional value and based on their features, as previously reviewed, algae as a potential functional additive in aquafeed is attracting increasing interest [100,101]. To date, a large number of alga species have been identified [36,102], representing a huge source of AMPs with different biological activities [10,13,14,33,34,35,36,37,40,99,102]; this means they also have great potential for the development of functional feeds [34,98,103,104,105,106,107]. One of the easier approaches is to include them as an ingredient in aquaculture feeds. However, several disadvantages have to be taken into consideration. First, the variability in nutrient composition is a major limiting factor for any practical mass utilization of algae in aquafeed and is mainly influenced by culture conditions [97,108]. Culture conditions determine the rate of secondary metabolite production, including AMPs [101], and their optimization could be a good way to enrich algae on AMPs for further biotechnological applications. Second, the cell wall composition and structure of AMPs influence their digestibility [109], which influences their release in the gut and biological activity. Although several strategies for increasing the digestibility of microalgae have been studied [110,111], a simple, economical, and cost-effective method has not yet been developed. The presence of anti-nutritive compounds in some algae species also needs to be taken into account when using them as feed additives [109]. Despite these disadvantages, the benefits in terms of the growth, welfare, and immune activity of several species of marine fish fed with aquafeed containing different amounts of microalgae have been described [112,113,114,115]. Other studies have tested the antimicrobial activity of several algae extracts [116,117,118,119]. However, as far as we know, no studies have identified the molecules involved or covered the effects of algal AMPs on immune responses in fish.

Furthermore, the use of algae as an ingredient in functional feeds would also be important in the implementation of the bioeconomic strategy of the European Union based on the growth and sustainable development of bio-sectors and value-added products of interest [29,120]. Thus, it will be necessary to determine the optimal industrial protocol for normal or AMP-enriched algae production and marine AMP isolation and characterization in order to design further biotechnological applications to cope with the actual challenges faced by the aquaculture sector, mainly the availability of raw materials, biosecurity and disease control, food quality and security, and environmental sustainability.

## 8. Conclusions and Future Perspectives

AMPs are interesting candidates for use in aquaculture as substitutes for antibiotics but also for antiviral, antifungal, or antiparasitic therapy to control infectious diseases. In addition, their immunostimulant effect and antioxidant activity and the lack of negative effects on fish and the environment make them even more powerful tools in aquaculture. Among the marine organisms that are rich sources of AMPs are algae, including cyanobacteria, microalgae, and macroalgae. Some species are currently being produced on an industrial scale and were previously used in the development of functional diets for some fish species in aquaculture. However, taking into account the wide variety of algae, culture species, and pathogens, further investigations are needed to identify and characterize algal AMPs and their application as an ingredient in functional diets or their isolation and subsequent use as pharmaceuticals.

## Figures and Tables

**Table 1:** Cyanobacterial peptides with potential for use in aquaculture.

Peptides	Sequences	Species	Activity	References
ND	LDAVNR MMLDF	*Arthrospira* *maxima*	Antiviral activity against COVID-19 by binding spike protein	[46]
	NALKCCHSCPA GVPMPNK IGP	*Arthrospira* *platensis*		
ND	KLENCNYAVELGK	*Limnospira maxima*	Antibacterial activity against Gram− *Escherichia coli* and Gram+ *Staphylococcus aureus*	[47]
Kawaguchipeptin A,B Cyclic peptides	WLNGDNNWSTP Kawaguchipeptin A: two prenylated tryptophans	*Microcystis aeruginosa* (NIES-88)	Antibacterial activity against Gram+ *Staphylococcus aureus*	[48]
Tenuecyclamide A–D Cyclic peptides	Tzl and mOzl rings Tenuecyclamide D: sulfoxide group	*Nostoc spongiaeforme* var. *tenue*	Antibacterial activity against Gram+ *Bacillus subtilis* and *Staphylococcus aureus*	[49]
Tolybyssidin A,B Cyclic peptides	FdhhaVT (acetate moiety) TVVPRLT VTITVVVFVdhhaRY	*Tolypothrix byssoidea* (EAWAG 195)	Antifungal activity against *Candida albicans*	[50]
Venturamide A,B Cyclic peptides	V(Tzl)A(Tzl)A(mOzl) T(Tzl)V(Tzl)A(mOzl)	*Oscillatoria* sp.	Antiparasitic activity against *Plasmodium falciparum*, *Trypanosoma cruzi*, and *Leishmania donovani* Weak toxicity against Vero cells	[51]
Aerucyclamide A-D Cyclic peptides	I(Tzl)T(mOzn)GCI(Tzn)C I(Tzl)T(mOzn)GCI(Tzl)C A(mOzn)TV(Ozl)SI(Tzl)C F(mOzn)TG(Tzn)CM(Tzl)C	*Microcystis aeruginosa* (PCC 7806)	Antiparasitic activity against *Plasmodium falciparum* and *Trypanosoma brucei rhodesiense*	[52,53]
AK-3 Cyclic peptide	IIEFAGGGKVMMY Most probable sequence: YGCMIFE	*Synechocystis* sp. (PCC 6803)	Antifungal activity against *Aspergillus niger*, *A. flavus*, and *A. fumigatus*	[54]
Aeruginazole A Cyclic peptide	N(Tzl)YL(Tzl)V(Tzl)FVGGG	*Microcystis* sp. (IL-323)	Antibacterial activity against Gram+ *Bacillus subtilis* No activity against Gram− *Escherichia coli* or Gram+ *Staphylococcus albus* Antifungal activity against *Saccharomyces cerevisiae* Cytotoxicity against human peripheral blood lymphocytes	[55]
Aeruginazole DA1497 Cyclic peptide	F(Tzl)GAIA(Tzl)SV(Tzl)PGVL(Tzl)PG	*Microcystis aeruginosa*	Antibacterial activity against Gram+ *Staphylococcus aureus*	[56]
ND	LDAVNR MMLDF	*Arthrospira* *maxima*	Anti-inflammatory activity by decreasing histamine release or producing reactive oxygen species (ROS) or modulating cytokine production depending on target cell type	[57,58]
ND	TDP(I or L)AAC(I or L)	*Arthrospira* sp.	Antibacterial and iron-chelating activity	[59]
Balgacyclamide A-B Cyclic peptides	V(mOzn)A(mOzn)I(Tzl) V(mOzn)TAI(Tzl)	*Microcystis aeruguinosa* (EAWAG 251)	Antiparasitic activity against *Plasmodium falciparum*, *Trypanosoma brucei rhodesiense*, and *Leishmania donovani*	[60]
SP-1	KLVDASHRLATGDVAVRA	*Arthrospira* *platensis*	Antibacterial activity against Gram− *Escherichia coli* and Gram+ *Staphylococcus aureus*	[61]
ND	PNN	*Arthrospira* *platensis*	Antioxidant activity through hydroxyl, superoxide, and 1,1′-diphenyl-2-picrylhydrazyl (DPPH) radical scavenging activity	[62]
FY11	FSESSAPEQHY Imidazole (intermediate biosynthesis) in histidine and pyrrolidine (secondary amine) ring in proline	*Arthrospira* *platensis*	Antioxidant activity through DPPH radical scavenging activity	[63]
LL12	LGLDVWEHAYYL	*Arthrospira* *platensis*	Antioxidant activity through hydroxyl, superoxide, DPPH, and 2,2′-azinobis-3-ethylbenzothiazoline-6-sulfonic acid (ABTS) or trolox equivalent antioxidant capacity (TEAC) radical scavenging activity and decreasing intracellular ROS upon induction of oxidative stress	[64]
ND	GMCCSR	*Arthrospira* *platensis*	Antioxidant activity through DPPH and ABTS radical scavenging activity, ferric reducing ability of plasma (FRAP), and protection against induced oxidative stress, proliferation activity, and collagen production	[65]
VH12	VKYVSPTCGPCH Imidazole in histidine and dithiol (-SH) active site in cysteine	*Arthrospira* *platensis*	Antioxidant activity through hydroxyl, superoxide, DPPH, and ABTS radical scavenging activity and decreasing ROS upon induction of oxidative stress	[66]
GM15	GGTCVIRGCVPKKLM Dithiol active site	*Arthrospira* *platensis*	Antioxidant activity through hydroxyl, superoxide, DPPH, and ABTS radical scavenging activity and decreasing ROS upon induction of oxidative stress Immunomodulatory activity by increasing granulocyte population	[67]
NL13	NPLSTQDDVAASL	*Arthrospira* *platensis*	Antioxidant activity through hydroxyl, superoxide, nitric oxide (NO), DPPH, and ABTS radical scavenging activity and decreasing ROS upon induction of oxidative stress Stimulation of cell migration and wound healing	[68]
ND	LAQELGSNR LGGEEVQEVLQQ ITGNASTIVSNAAR APYDESEIAFH APLDESEMAFH VTAGLVGGGAGK	*Arthrospira* *platensis*	Antioxidant activity through hydroxyl and ABTS radical scavenging activity Antibacterial and iron-chelating activity	[69]
ND	FFEFF EYFDALA VTAPAASVAL	*Arthrospira* *platensis*	Antioxidant activity through DPPH and ABTS radical scavenging activity, decreasing hemolysis and malondialdehyde (MDA), and increasing antioxidant enzyme activity upon induction of oxidative stress	[70]
NV-14	NVRIGAGSVVLRDV	*Arthrospira* *platensis*	Antioxidant activity through hydroxyl, superoxide, hydrogen peroxide (H_2_O_2_), NO, DPPH, and ABTS radical scavenging activity and protecting against induced oxidative stress	[71]

ND, not defined.

**Table 2:** Microalgal peptides with potential for use in aquaculture.

Peptides	Sequences	Species	Activity	References
AQ-1766 H	LWFYTMW	*Tetraselmis* *suecica*	Antibacterial activity against Gram− *Escherichia coli*, *Salmonella typhimurium*, and *Pseudomonas aeruginosa* and Gram+ *Bacillus cereus*, methicillin-resistant *Staphylococcus aureus*, *Micrococcus luteus*, and *Listeria monocytogenes*	[72]
Alanine and lysine synthetic analogs of AQ-1766	**A**WFYTMWH LWF**A**TMWH LWFY**A**MWH LWFYT**A**WH **K**WFYTMWH LWF**K**TMWH LWFY**K**MWH LWFYT**K**WH	*Tetraselmis* *suecica*	Synthetic alanine and lysine synthetic enhances antibacterial activity of AQ-1766; lysine replacement causes a change of beta turn tendency to helical trend	[72]
APWP	VECYGPNRPQF	*Chlorella* *vulgaris*	Antioxidant activity through hydroxyl, superoxide, DPPH, ABTS, and peroxyl radical scavenging activity and anti-inflammatory activity through decreasing levels of adhesion molecules and chemokines	[73,74,75,76]
CDP (Chlorella-derived peptide)	ND	*Chlorella* sp.	Anti-UVB and UVC irradiation damage through regulating specific gene expression	[77]
		*Chlorella* *pyrenoidosa*		[78]
ND	MPGPLSPL	*Pavlova* *lutheri*	Involved in myofibroblast differentiation	[79]
ND	LNGDVW	*Chlorella* *ellipsoidea*	Antioxidant activity through hydroxyl, peroxyl, and DPPH radical scavenging activity Enhanced cell viability against induced cytotoxicity by decreasing sub-G1 hypodiploid cells and apoptotic body formation	[80]
NIPP-1 NIPP-2	PGWNQWFL VEVLPPAEL	*Navicula* *incerta*	Protection against ethanol-induced cytotoxicity and hepatic fibrosis Antioxidant activity by increasing glutathione (GSH) levels and decreasing gamma-glutamyltransferase (GGT) activity Anti-inflammatory activity by regulating cytokine levels	[81,82]
ND	MPDW	*Nannochloropsis* *oculata*	Promotion of osteoblast differentiation	[83]
ND	MGRY	*Pavlova* *lutheri*	Antioxidant activity through hydroxyl, H_2_O_2_, and DPPH radical scavenging activity and decreasing ROS production Modulation of inflammatory response by activating some elements of extracellular signal kinase pathways	[84]
ND	WPRGYFL SDWDRF	*Tetradesmus* *obliquus*	Antioxidant activity through DPPH and ABTS radical scavenging activity	[85]
ND	ILTKAAIEGK IIYFQGK NDPSTVK TVRPPQG	*Dunaliella* *salina*	Antioxidant activity through DPPH radical scavenging activity	[86]
Coco1 Coco2 Coco3	FTILKKLKSFIK KLVKKLLKKYITF LARFVLRILKYGFK	*Coccomyxa* *subellipsoidea* (C-169)	Antibacterial activity against Gram− *Pseudomonas aeruginosa* (coco1 and coco2), *Escherichia coli*, *Acinetobacter baumannii*, *Pseudomonas aeruginosa*, and *Klebsiella pneumoniae* and Gram+ *Staphylococcus aureus* (coco3)	[87]

ND, not defined.

**Table 3:** Macroalgal peptides with potential for use in aquaculture.

Peptides	Sequences	Species	Activity	References
SECMA1	EDRLKP	*Ulva* sp.	Increase production of proteoglycans and glycosaminoglycans	[88]
ND	IRLIIVLMPILMA PAIA FPAI ILMA VFPAIAM AQILP NIGK	*Palmaria* *palmata*	Anti-inflammatory activity	[89]
ND	TITLDVEPSDTIDGVK ISGLIYEETR MALSSLPR ILVLQSNQIR ISAILPSR IGNGGELPR LPDAALNR EAESSLTGGNGCAK QVHPDTGISK	*Saccharina* *longicruris*	Antibacterial activity against Gram+ *Staphylococcus aureus*	[90]
PYP1 / PY-PE	ALEGGKSSGGGEATRDPEPT	*Pyropia* *yezoensis*	Proliferation activity and stimulation of cell growth Immunomodulatory activity by regulating protein kinase signaling pathways and cellular cycle	[91,92]
PPY1	KAQAD	*Pyropia* *yezoensis*	Antioxidant activity by inhibiting release of ROS and NO intermediate in activated macrophages and anti-inflammatory activity by regulating cytokine and protein kinase signaling pathways	[93]
ND	FITDGNK NAATIIK ANAATIIK SDITRPGGQM DNIQGTKPA LITGA LITGAA LITGAAQA LGLSGK LTIAPK ITLAPK ITIAPK VVPT QARGAAQA	*Palmaria* *palmata*	Antioxidant activity through FRAP and oxygen radical absorbance capacity (ORAC)	[94]
PBP1 PBP2 PBP3 PBP4 PBP5 PBP6 PBP7 PBP8 PBP9 PBP10 PBP11 PBP12 PBP13	KAAAVAFITNTASQRK RYVSYALLAGDPSVLEDRC MQDAITSVINAADVQGKY RAAATIAANAATIIKE RYATYGMLAGDPSILEERV RLVTYGIVAGDVTPIEEIGLVGVKE RFPSSSDLESVQGNIQRA KSVITTTISAADAAGRFPSSSDLESVQGNIQRA RTLNLPTSAYVASFAFARD RFLSNGELQAINGRY RLITGAAQSVYTKF KTPITEAIASADSQGRF KFPYVTQMPGPTYASSAIGKA	*Pyropia* *yezoensis*	Antioxidant activity against induced oxidative stress in human cells, decreasing ROS and increasing antioxidant enzyme activity and expression	[95]
ND	DYYLR AGFY YLVA AFIT MKTPITE TYIA LDLW	*Porphyra* *dioica*	Antioxidant activity through enhanced ORAC upon gastrointestinal digestion	[96]
ND	ELWKTF	*Gracilariopsis* *lemaneiformis*	Antioxidant activity through DPPH radical scavenging activity	[97]

ND, not defined.

## Data Availability

Data sharing not applicable.
